# Retraction: Clonal Analysis of Meningococci during a 26 Year Period Prior to the Introduction of Meningococcal Serogroup C Vaccines

**DOI:** 10.1371/journal.pone.0141008

**Published:** 2015-10-16

**Authors:** Christopher B. Sullivan, Mathew A. Diggle, Robert L. Davies, Stuart C. Clarke

The authors wish to retract this article at the request of the Scottish Haemophilus Legionella Meningococcus Pneumococcus Reference Laboratory (SHLMPRL). Although the work was part of an independently-funded research grant awarded to Stuart Clarke, Matthew Diggle and Robert Davies by the Scottish Executive, they did not have permission from the current SHLMPRL management to publish the data in their current form. The anonymised data in the MLST database will remain available. The authors apologise to the readers.
